# Cultivation of the microalgae *Chlamydomonas reinhardtii* and *Desmodesmus quadricauda* in highly deuterated media: Balancing the light intensity

**DOI:** 10.3389/fbioe.2022.960862

**Published:** 2022-09-05

**Authors:** Veronika Kselíková, Kamila Husarčíková, Peter Mojzeš, Vilém Zachleder, Kateřina Bišová

**Affiliations:** ^1^ Laboratory of Cell Cycles of Algae, Centre Algatech, Institute of Microbiology of the Czech Academy of Sciences, Třeboň, Czechia; ^2^ Faculty of Science, University of South Bohemia, České Budějovice, Czechia; ^3^ Faculty of Food and Biochemical Technology, University of Chemistry and Technology Prague, Prague, Czechia; ^4^ Institute of Physics, Faculty of Mathematics and Physics, Charles University, Prague, Czechia

**Keywords:** microalgae, deuterium, light intensity, cell division, deuterated compounds, physical stress

## Abstract

The production of organic deuterated compounds in microalgal systems represents a cheaper and more versatile alternative to more complicated chemical synthesis. In the present study, we investigate the autotrophic growth of two microalgae, *Chlamydomonas reinhardtii* and *Desmodesmus quadricauda*, in medium containing high doses of deuterated water, D_2_O. The growth of such cultures was evaluated in the context of the intensity of incident light, since light is a critical factor in the management of autotrophic algal cultures. Deuteration increases the light sensitivity of both model organisms, resulting in increased levels of singlet oxygen and poorer photosynthetic performance. Our results also show a slowdown in growth and cell division processes with increasing D_2_O concentrations. At the same time, impaired cell division leads to cell enlargement and accumulation of highly deuterated compounds, especially energy-storing molecules. Thus, considering the specifics of highly deuterated cultures and using the growth conditions proposed in this study, it is possible to obtain highly deuterated algal biomass, which could be a valuable source of deuterated organic compounds.

## Introduction

Algal biomass is a source of numerous valuable compounds such as lipids, carbohydrates or carotenoids that are used in biotechnology to produce biofuel (Brennan and Owende, 2010), biopolymers (Brennan and Owende, 2010; Madadi et al., 2021), animal feed (Becker, 2007) or dietary supplements (Eriksen, 2016; Deeba et al., 2020). The compounds can be further exploited by producing their stable-isotope-labelled counterparts, which is possible by cultivating microalgae on labelled substrates (Gireesh et al., 2001; Bhosale et al., 2006; Saha et al., 2013; Zachleder et al., 2018). Due to the photoautotrophic nature of microalgae, cheap inorganic labelled substrates are sufficient to produce various labelled biomolecules with high added value, making production economically viable. One specific application of this approach is the production of highly deuterated biomolecules in microalgae. Deuterium is a stable isotope of hydrogen with an extra neutron in its nucleus. Compounds labelled with deuterium, along with other isotopically labelled molecules, are commonly used as tracers in ecology, proteomics, and metabolomics (Lehmann, 2016; Zachleder et al., 2018) or in pharmacology to produce deuterated drugs (Kushner et al., 1999; Cargnin et al., 2019). The production of deuterated biomolecules, such as fatty acids or pigments, using microalgae has been achieved in several microalgal species, including *Chlorella* spp. (Delente, 1987; Gireesh et al., 2001; Bhosale et al., 2006). The percentage of deuterium enrichment ranged from 25% in deuterated eicosapentaenoic acid, chlorophyll *b*, lutein, astaxanthin, or β-carotene produced by two biotechnologically important green algae *Haematococcus pluvialis* and *Phaeodactylum tricornutum* (Hattori et al., 1965; Crespi et al., 1968) up to 50% in chlorophyll *a* produced by cyanobacterium *Halomicronema hongdechloris* (Garg et al., 2017) and 58% in lutein produced by *Chlorella protothecoides* (Bhosale et al., 2006)*.* The deuterated compounds produced by microalgae are more valuable than their protonated variants, but their production is fraught with difficulties. Replacement of protium (^1^H) with deuterium (^2^H or D) elicits a kinetic isotope effect (KIE). This is described as a change in the reaction rates of isotopically substituted atoms or molecules (Bigeleisen and Mayer, 1947). The KIE is a cause of discrimination between lighter and heavier isotopes, as observed in a variety of biochemical reactions, e.g., the well-documented discrimination of RubisCO regarding use of ^14^C in carbon fixation (von Caemmerer et al., 1997; Kubásek et al., 2013). The isotopic exchange between hydrogen and deuterium exhibits the largest KIE among the stable isotopes of all bioelements, which is due to the large mass differences between hydrogen and deuterium (Wade, 1999). The strong KIE caused by deuterium causes numerous perturbations in cell physiology. The most prominent effects of deuteration are the triggering of a general stress response and the reduction of cell growth and rates of division as already published in several microalgae, including *Chlorella* spp. or *Chlamydomonas* sp. (Unno et al., 1987; Kollars and Geraets, 2008; Zachleder et al., 2021a; Kselíková et al., 2021). Therefore, understanding the stressful effect of deuterium in microalgae is a prerequisite for successful cultivation of microalgae on highly deuterated substrates. Describing the cellular response to deuterium-induced stress will enable an understanding of the consequences of KIE in complex biological systems. This knowledge could then be used to optimize culture management of deuterated cultures. However, little attention was paid to specific changes in cell cycle progression of deuterated cultures so far. At the same time, necessary alterations of cultures conditions induced by deuterium treatment remains understudied as well.

Light is a critical abiotic factor for microalgal growth, especially in photoautotrophic cultures where microalgae rely on light as the sole source of energy for carbon fixation. Available light can be characterized from two main perspectives - by its quality and quantity. The quality of light describes its spectral composition, with photosynthetically active radiation (PAR) typically between 400 and 700 nm, corresponding to the absorption of the major plant/algal pigments. Light quality, particularly the proportion of red and blue light, affects growth and cell cycle progression (Oldenhof et al., 2004; Li et al., 2021) and could be used in biotechnology to promote the production of valuable compounds such as lipids (Shu et al., 2012; Chavez-Fuentes et al., 2018) or carotenoids (Katsuda et al., 2004; Fu et al., 2013; Lee et al., 2018) in microalgae. The amount of light is usually expressed as light intensity, although this term can correspond to several metrics with different definitions. In algal cultivation, it usually refers to photosynthetic photon flux density (PPFD) in μmol photons·m^−2^⋅s^−1^, i.e., the amount of photosynthetically active photons incident on a surface per unit time. PPFD is a relevant way to express light intensity in algal cultures, since it represents only the photosynthetically active radiation available in the area of interest (the surface of the bioreactor, etc.). In general, increasing light intensity increases biomass productivity; however, excess light can cause stress and lead to photoinhibition, which limits further biomass production (Amini Khoeyi et al., 2011; Difusa et al., 2015; Zachleder et al., 2021a).

The present work describes the growth of the microalgae *Chlamydomonas reinhardtii* and *Desmodesmus quadricauda* exposed to light of different intensities and highly deuterated media. The effects of light intensity and deuterium content in culture medium are discussed individually and in combination. Particular attention is given to the characterization of cell growth and cell division, as these processes are sensitive to the two factors discussed. Both *C. reinhardtii* and *D. quadricauda* are well-established model organisms (Harris, 2001; Bisova and Zachleder, 2014) that can also be used in algal biotechnology thanks to their rapid growth and well-characterized biology. They both divide by multiple fission, a division pattern that allows the formation of more than two daughter cells within a cell cycle. The number of daughter cells formed is within a species-specific range and is largely influenced by external growth conditions, such as light intensity and temperature (Bisova and Zachleder, 2014; Zachleder et al., 2021a; Zachleder et al., 2021b). Consequently, microalgae that divide by multiple fission show considerable plasticity in response to changing environmental conditions, allowing for better monitoring and descriptions of microalgal responses to such treatments.

## Materials and methods

### Model organism and culture conditions

**FIGURE 1:**
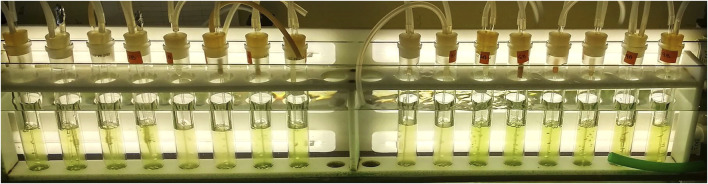
Cylindrical glass bioreactors in a temperature-controlled water bath illuminated by fluorescent tubes. Cultures are mixed by bubbling from the bottom of the bioreactors.

Two microalgae were used in this study: *Chlamydomonas reinhardtii* strain 21gr (CC-1690; Chlamydomonas Genetics Center, Duke University, Durham, NC, United States) and *Desmodesmus quadricauda* strain Greifswald/15 (CCALA strain number 463; CCALA, Czech Academy of Sciences, Třeboň, Czech Republic). Cultures were maintained in cylindrical glass bioreactors (inner diameter 30 mm, height 200 mm, effective volume 100 ml), placed in a temperature-controlled water bath (30°C ± 0.5°C) and illuminated from one side with dimmable incandescent lamps (Dulux L55W/950 Daylight, Osram, Munich, Germany). Cultures were mixed by bubbling 2% CO_2_ in air (v/v) through a thin glass tube from the bottom of the cylinder (Figure 1).

All experiments were performed with synchronous cultures, i.e., cultures, in which all cells are at approximately the same stage of their cell cycle. Such cultures were prepared using light-dark synchronization protocols (Hlavová et al., 2016). Briefly, cultures were inoculated into fresh medium from a plate and grown at 30°C and an incident light intensity of 500 μmol photons·m^−2^⋅s^−1^ at the surface of the cultivation vessel. The light-dark regime was set to 13 h of light and 11 h of darkness for *C. reinhardtii* and 15 h of light and 9 h darkness for *D. quadricauda* under these conditions. Cell density was maintained by dilution to 1×10^6^ cells mL^−1^ at the beginning of each light cycle. Cultures were maintained under the same conditions for at least three consecutive cycles to obtain highly synchronous cultures. Synchronous cultures consisting of small daughter cells were centrifuged at 3000 × g for 5 min and re-suspended in desired growth medium with/without D_2_O to the initial cell density before the start of light period. Growth conditions during the experiment were the same as described above for synchronization, except for the incident light intensity, which was set to 100/200/400 μmol photons·m^−2^⋅s^−1^ continuous light measured with a ULM-500 light meter equipped with a spherical microquantum sensor US-SQS/L (Walz, Effeltrich, Germany) placed in the centre of the cultivation vessel. The sensor provides a reliable measurement of the scattered light and the values obtained better represent the light available to the cultures in the cylindrical vessel. No dark periods were used during the course of the experiments. To prevent the light available to individual cells decreasing with increasing cell density, cultures were maintained in a semi-batch mode controlled by the optical density measured at 750 nm (OD_750_), i.e., when cultures reached an OD_750_ of 0.4, they were diluted with fresh medium to OD_750_ = 0.1 to ensure an approximately constant supply of light to individual cells. The duration of the experiment (102 h) was set to follow a complete cell cycle in the slowest monitored culture (see below).

### Growth medium

*C. reinhardtii* was cultured in high salt (HS) medium (Sueoka, 1960) with modifications described by Hlavová et al. (2016); *D. quadricauda* was cultured in Šetlík’s Simmer (ŠS) medium of half concentration as previously described (Hlavová et al., 2016). Both media do not contain any carbon source and therefore only support autotrophic growth. The media were prepared using distilled water or deuterated (heavy) water (deuterium oxide, D_2_O; 99.95 atom%, catalog number 300101500, Silantes, Munich, Germany). For the preparation of deuterated medium, all stock solutions were pre-dried to obtain the required amount of mineral salts, which were re-suspended in a mixture of normal and deuterated water to obtain complete mineral medium with 70% or 90% D_2_O.

### Evaluation of growth and division

Cultures were sampled every 6 h. Culture growth was measured as optical density at 750 nm (OD_750_). For cell count analysis, 1 ml of the culture was fixed with 100 μL of 2.5% glutaraldehyde and stored at 4°C until analysis. Cell numbers of *C. reinhardtii* were analyzed using a Beckman Coulter Multisizer 4 (Beckman Coulter Life Sciences, Brea, CA, United States) by diluting 50 µL of the fixed cell suspension in 10 ml of 0.9% NaCl (*w/v*) electrolyte solution. Cells of *D. quadricauda* were counted manually using a Bürker chamber, as the presence of multicellular coenobia prevented accurate cell counting using the Multisizer 4. The same principle was applied to *C. reinhardtii* when cells formed clusters that prevented accurate machine counting. Cell division in synchronous culture was monitored by light microscopy, using fresh samples at regular intervals.

### Fv/Fm measurement

The quantum yield of photosynthesis was measured as the F_V_/F_M_ ratio of chlorophyll fluorescence. Aliquots of 2 ml were taken from the culture and darkened for 30 min in 10 mm × 10 mm plastic cuvettes at room temperature. The cell suspension was mixed gently by inverting the cuvettes several times before measurement. Quantum yield was measured using an Aqua-Pen-C 100 (Photon Systems Instruments, Drasov, Czech Republic) set according to the manufacturer’s instructions.

### Light and fluorescence microscopy

At each sampling time point, 1 ml of culture was centrifuged at 3000 × g for 3 min and frozen at −20°C until analysis. Freshly thawed pellets were stained with 5 μg ml^−1^ of 4,6ʹ-diamidino-2-phenylindole (DAPI) in S buffer (Kuroiwa and Suzuki, 1980) and incubated at room temperature for 20 min in the dark to visualize cell nuclei. The stained cells were observed using a microscope (model BX51, Olympus, Tokyo, Japan) equipped with a U-MWIBA2 filter block (excitation: 460–490 nm, emission: 510–550 nm). To visualize the starch granules, cells were stained with Lugol’s solution (1 g I, 5 g KI, 100 ml H_2_O) at a final concentration 0.5 μL Lugol’s solution per 20 μL cell suspension directly on the slide. Photomicrographs were taken with a DP72 camera (Olympus, Tokyo, Japan).

### Confocal Raman microscopy

Raman mapping was done using a confocal Raman microscope (WITec alpha300 RSA, Germany) equipped with a ×60 water-immersion UPlanSApo objective (Olympus, Japan). A 532 nm laser with a power of approximately 20 mW at the focal plane was used. To decrease the persistent fluorescence background of the impaired photosynthetic apparatus of the fixed cells, pigments (chlorophyll, carotenoids) were washed out by methanol for 30 s. After the methanol-washing, the cells were harvested by centrifugation (3000 × g, 30 s), the pellet was resuspended in deionized water and the cells were twice rinsed to remove traces of methanol. We have found that such a methanol-washing removes only pigments and neutral lipids leaving starch, crystalline guanine, polyphosphate, and remains of plastids in the cells. The 5 μL of the resulting cell suspension was mixed with 5 μL of 1% low-temperature-melting agarose and spread under a 20 mm diameter, 0.18 mm-thick quartz coverslip sealed with CoverGrip (Biotium, United States). Raman measurements were performed with a scanning step of 200 nm in both directions, voxel size 1 μm^3^, and an integration time of 0.07 s per voxel. On average, 5–10 cells of each strain from each cell culture were measured. Standards of pure chemical substances (deuterated starch, deuterated d5-guanine, polyphosphate) were measured in water suspension. Data was analyzed using WITec Project SIX Plus v6.0 software (WITec, Germany) to implement the following steps: cosmic ray removal, background subtraction, cropping of the spectral edges affected by detector margins, spectral unmixing with the True Component Analysis tool, and averaging of the mean spectra, summarizing multiple measurements in order to optimize the signal-to-noise ratio.

### Estimation of oxidative stress

Oxidative stress in each experimental treatment was estimated using the commercial Singlet Oxygen Sensor Green probe (catalog number S36002, Thermo Fisher Scientific, Waltham, MA, United States), which measures the relative amount of singlet oxygen. For analysis, 100 μL of fresh culture was pipetted into a 96-well plate and combined with 5 μL of 0.1 mM Singlet Oxygen Sensor Green in 100% (v/v) methanol. The blank sample consisted of 100 μL of culture combined with 5 μL of 100% (v/v) methanol. The specific signal was measured using an Infinite 200 PRO microplate reader (Tecan, Männedorf, Switzerland) equipped with an excitation filter of 485 nm and an emission filter of 535 nm. The fluorescence intensity of the samples was normalized against the blank value.

### Statistics

All experiments were performed in three biological replicates (*n* = 3). Presented results are the average and standard deviations from all three replicates. MS Excel 2010 (Microsoft) with Real Statistics Resource Pack (https://www.real-statistics.com/free-download/real-statistics-resource-pack/, accessed on 5 April 2022) was used to generate statistics. Comparison of mass, cell doubling times, and singlet oxygen levels between experimental treatments was performed using the two-way ANOVA test and Tukey’s HSD test. A p-value < 0.05 was considered significant.

## Results

### Growth and division

**FIGURE 2:**
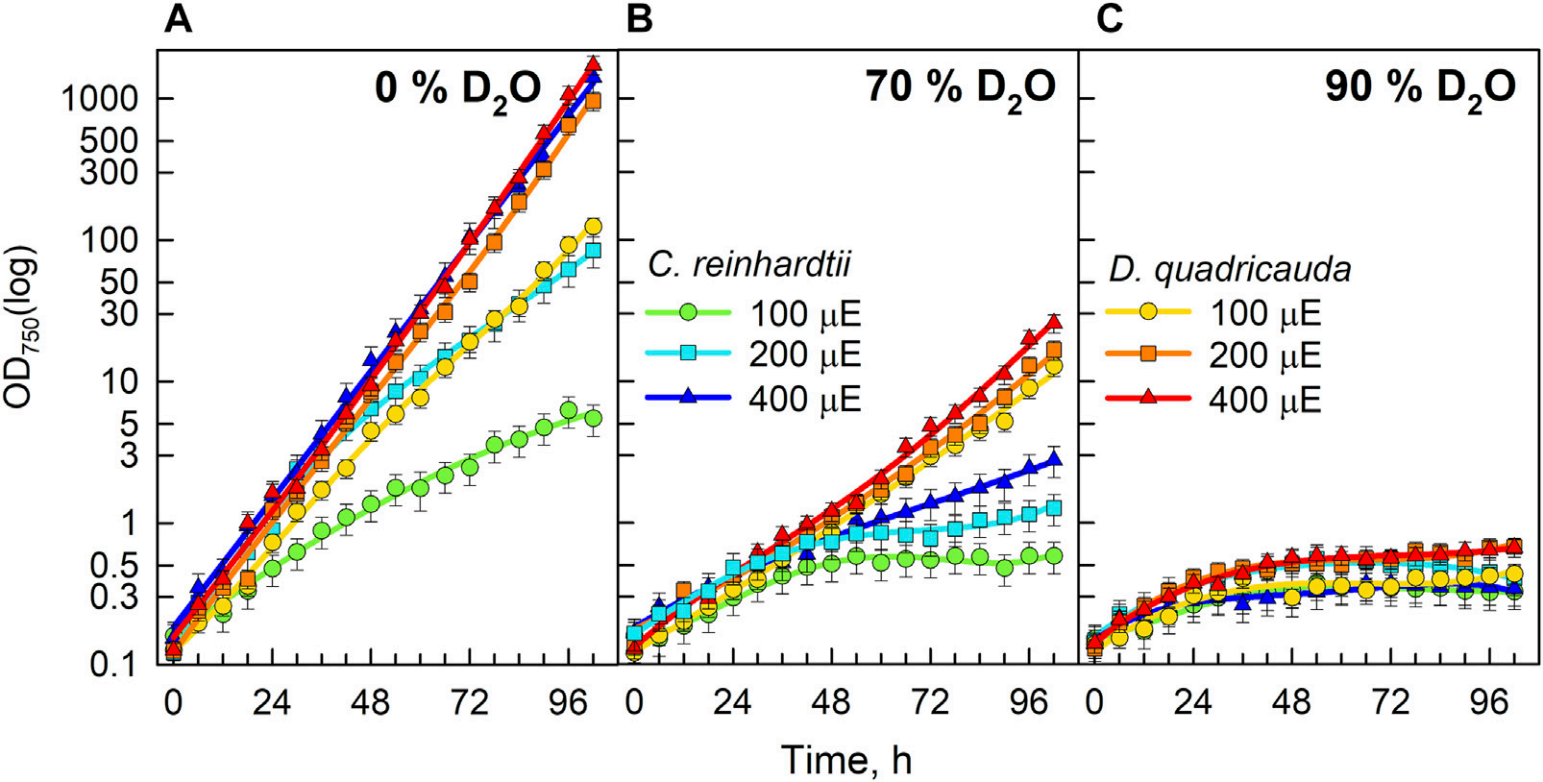
Growth of algal cultures measured as optical density at 750 nm (OD_750_) in control culture in 0% D_2_O **(A)**, 70% D_2_O **(B)** or 90% D_2_O **(C)** at three light intensities 100, 200, and 400 μmol m^−2^ s^−1^ extrapolated from semi-batch cultivation mode. Light intensities are referred to in μE (instead of μmol photons·m^−2^⋅s^−1^), in order to fit the legends. Note logarithmic scale on Y axis. Although the cultures were regularly diluted to ensure a constant light supply (semi-batch cultivation), the values obtained were recalculated using individual dilution factors. Therefore, the graphs represent a projection of growth in semi-batch cultivation mode to continuous growth. In this way, the graphs are easier to read and compare.

The microalgae *Chlamydomonas reinhardtii* and *Desmodesmus quadricauda* were cultured in growth media containing 0%, 70% or 90% D_2_O at incident light intensities of 100, 200, or 400 μmol photons·m^−2^⋅s^−1^. The cultivation conditions were chosen to cover as wide a range of conditions as possible. The two D_2_O concentrations represent 1) a relatively high concentration with little effect on algal growth and division, and 2) a very high concentration with profound effects on algal physiology but still allowing some growth. Light intensities ranged from low to relatively high light levels: 1) the lowest light intensity represented the lowest light intensity that allowed some growth, 2) the highest was chosen so that the combined effect of stress from high light and high D_2_O concentration did not result in immediate cell death. As measured by optical density at 750 nm (OD_750_), the control culture of *D. quadricauda* in 0% D_2_O grew comparably at both 200 μmol photons·m^−2^⋅s^−1^ and 400 μmol photons·m^−2^⋅s^−1^ but significantly slower at 100 μmol photons·m^−2^⋅s^−1^ (Figure 2A). The control culture of *C. reinhardtii* at 400 μmol photons·m^−2^⋅s^−1^ grew comparably to the control culture of *D. quadricauda* at the same incident light intensity, whereas the culture of *C. reinhardtii* at 200 μmol photons·m^−2^⋅s^−1^ grew the same as that of *D. quadricauda* at 100 μmol photons·m^−2^⋅s^−1^. The culture of *C. reinhardtii* at 100 μmol photons·m^−2^⋅s^−1^ showed the slowest growth of all control cultures (Figure 2A). Cultures growing in medium containing 70% D_2_O grew significantly slower than the controls at all light intensities tested (Figure 2B). Cultures of *D. quadricauda* in 70% D_2_O showed comparable growth regardless of light intensity, but grew more slowly than any *D. quadricauda* culture in control medium (Figure 2, compare A and B). *C. reinhardtii* also grew more slowly in 70% D_2_O than in any of the control cultures, but there was a clear effect of light intensity, with higher light intensity resulting in faster growth (Figure 2B). Cultures of *D. quadricauda* and *C. reinhardtii* in 90% D_2_O grew significantly slower than in lower D_2_O concentrations, reaching a maximum OD_750_ that was approximately twice the initial value for both *D. quadricauda* and *C. reinhardtii* (Figure 2C).

**FIGURE 3:**
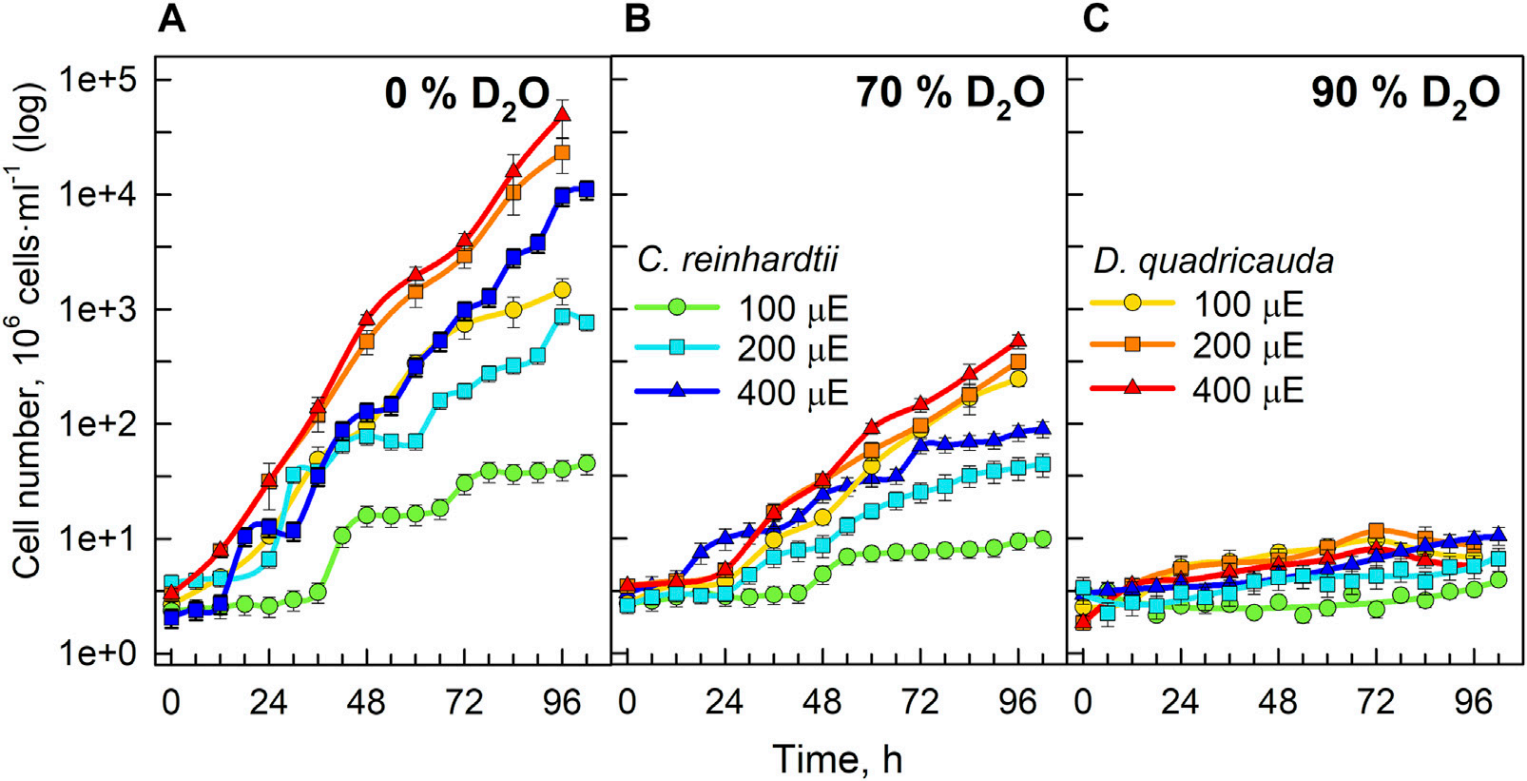
Cell numbers of cultures grown in 0% D_2_O **(A)**, 70% D_2_O **(B)** or 90% D_2_O **(C)** at three light intensities (100, 200, and 400 μmol m^−2^ s^−1^) extrapolated from semi-batch cultivation mode. Light intensities are described in μE (instead of μmol photons·m^−2^⋅s^−1^), in order to fit the legends. Note the logarithmic scale on the Y axis. Although the cultures were regularly diluted to ensure a constant light supply (semi-batch cultivation), the values obtained were recalculated using individual dilution factors. Therefore, the graphs represent a projection of growth in semi-batch cultivation mode to continuous growth. In this way, the graphs are easier to read and compare.

Since the OD_750_ reflects the growth of the culture, the increase in OD_750_ could be caused by an increase in cell number, an increase in cell volume, or their combination. The changes in cell numbers of *D. quadricauda* and *C. reinhardtii* cultures grown in different D_2_O concentrations and incident light intensities are shown in Figure 3. Cell numbers in the control cultures of *D. quadricauda* increased exponentially at both 400 μmol photons·m^−2^·.s^−1^ and 200 μmol photons·m^−2^⋅s^−1^; at 100 μmol photons·m^−2^⋅s^−1^, the increase in cell number was similar but slightly slower, reflecting the trends observed for OD_750_ (Figure 3A). Cell numbers in *C. reinhardtii* control cultures showed a clear response to incident light intensity, with higher light intensities producing more cells. The synchrony of the cultures and the sampling interval also allowed individual divisions to be seen as a sharp increase in cell numbers during the experiment (Figure 3A). The control culture of *C. reinhardtii* at 100 μmol photons·m^−2^⋅s^−1^ performed two complete cell divisions, the first after approximately 35 h, and the second after 70 h. The same culture at 200 μmol photons·m^−2^⋅s^−1^ performed a total of 3 cell cycles, each about 27 h apart. At the highest light intensity, the first two divisions were about 18 h apart. Subsequent divisions were more difficult to distinguish because the degree of synchrony in the continuously illuminated culture decreases with time (Figure 3A). Cultures of *D. quadricauda* grown in 70% D_2_O achieved significantly lower cell numbers than in the control culture regardless of incident light intensity. Cultures of *C. reinhardtii* in 70% D_2_O at 400 μmol photons·m^−2^⋅s^−1^ and 200 μmol photons·m^−2^⋅s^−1^ attained cell numbers comparable to those of the control culture at 100 μmol photons·m^−2^⋅s^−1^. From the course of cell number increase, we can conclude that *C. reinhardtii* in 70% D_2_O completed the first cell cycle after approximately 48 h at 100 μmol photons·m^−2^⋅s^−1^, after 35 h at 200 μmol photons·m^−2^⋅s^−1^ and after 23 h at 400 μmol photons·m^−2^⋅s^−1^ (Figure 3B). The course of subsequent cell divisions was more difficult to determine (see above). All cultures grown in 90% D_2_O barely increased their cell number during the experiment and reached a cell number about twice as high as the initial value (Figure 3C).

**FIGURE 4:**
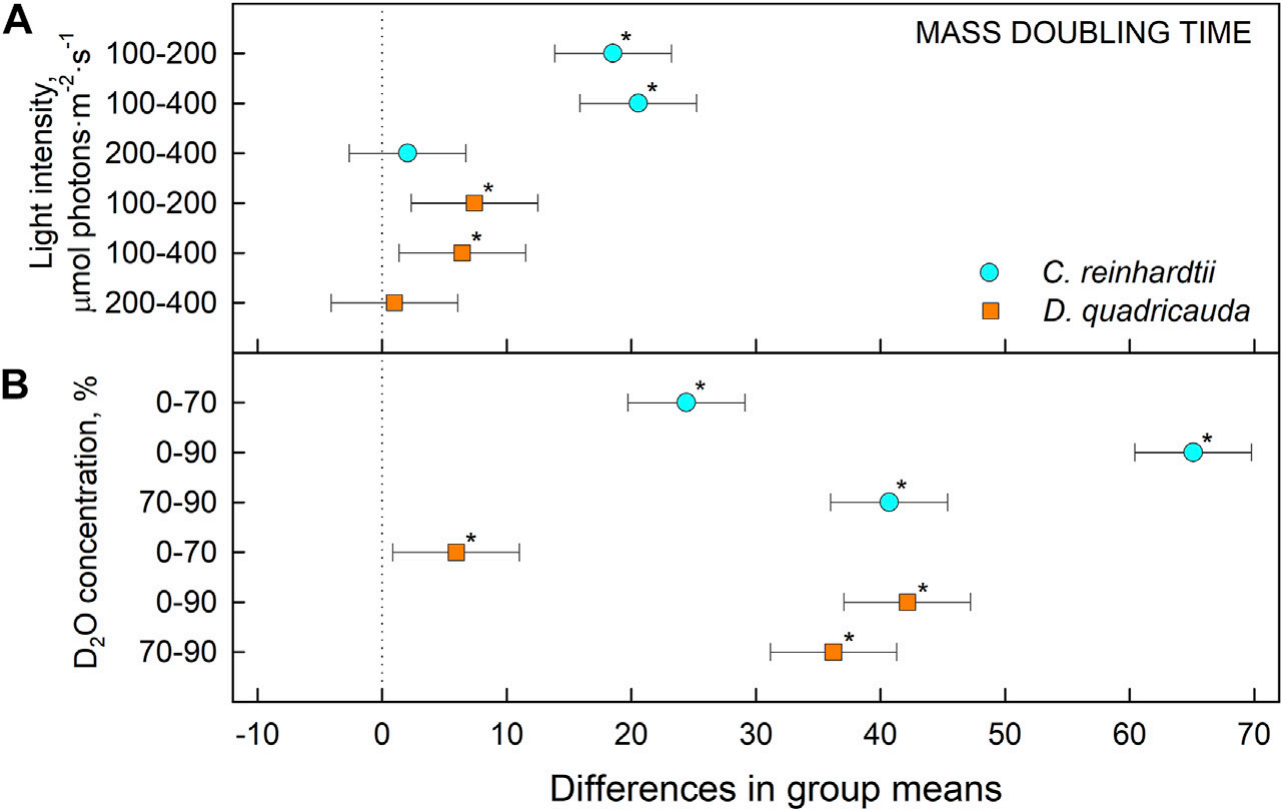
Tukey’s HSD test of mass doubling times plotted as 95% confidence intervals of mean difference between groups indicated on the left. The effect of light intensity **(A)** and the effect of D_2_O concentration **(B)** on mass doubling times of *C. reinhardtii* (cyan circles) and *D. quadricauda* (orange squares) are plotted separately. A difference in group means (X axis) equal to zero indicates that the group means are equal, so only a confidence interval that does not contain zero is statistically significant (*p* = 0.05, indicated by an asterisk).

**FIGURE 5:**
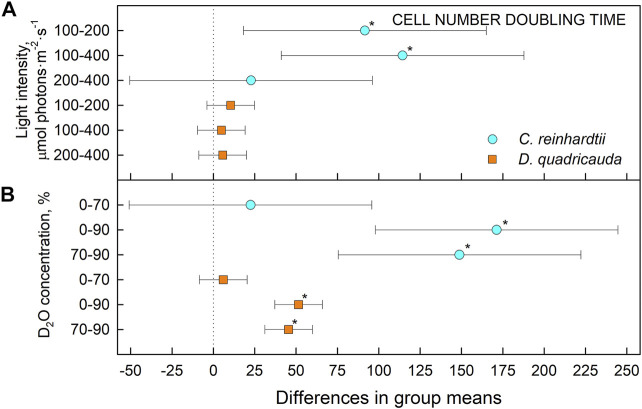
Tukey’s HSD test of cell number doubling times plotted as 95% confidence intervals of mean difference between groups indicated on the left. The effect of light intensity **(A)** and the effect of D_2_O concentration **(B)** on mass doubling times of *C. reinhardtii* (cyan circles) and *D. quadricauda* (orange squares) are plotted separately. A difference in group means (X axis) equal to zero indicates that the group means are equal, so only a confidence interval that does not contain zero is statistically significant (*p* = 0.05, indicated by an asterisk).

To quantify culture growth, we calculated doubling times for each experimental treatment based on OD_750_ (Figure 2) and cell numbers (Figure 3), expressing doubling times for mass and cell number, respectively (Table 1). The general trend of faster culture growth, both in terms of mass and cell numbers, at higher light intensities (seen as a decrease in doubling times) was disrupted at the highest D_2_O concentration, as increasing the light intensity above 200 μmol photons·m^−2^⋅s^−1^ did not result in a significant decrease in doubling times for either *D. quadricauda* or *C. reinhardtii*. On the other hand, an increase in doubling times, both in terms of mass and cell numbers, was observed with increasing D_2_O concentrations at all light intensities tested. Two-way analysis of variance (ANOVA) was performed to analyze the effects of light intensity and D_2_O concentration on mass and cell number doubling times for both *D. quadricauda* and *C. reinhardtii*. It revealed that there was a statistically significant interaction between the effects of light intensity and D_2_O concentration on mass doubling time for *C. reinhardtii* [F (4, 18) = 5.531, *p* = 0.004] as well as for *D. quadricauda* [F (4,18) = 4.695, *p* = 0.009]. A simple main effects analysis showed that both light intensity and D_2_O concentration had a statistically significant effect on mass doubling times in *C. reinhardtii* (both with *p* < 0.001) and *D. quadricauda* (*p* = 0.003 and *p* < 0.001, respectively). In addition, a two-way ANOVA revealed a statistically significant interaction between the effects of light intensity and D_2_O concentration on cell number doubling time in *C. reinhardtii* [F (4, 18) = 6.145, *p* = 0.003], but not in *D. quadricauda* [F (4,18) = 1.369, *p* = 0.284]. A simple main effects analysis showed that both light intensity and D_2_O concentration had a statistically significant effect on *C. reinhardtii* mass doubling times (*p* = 0.002 and *p* < 0.001, respectively). To evaluate the differences between each experimental treatment in terms of mass and cell doubling times, Tukey’s HSD test for multiple comparisons was performed for all light intensities and D_2_O concentrations tested. The results, presented as 95% confidence intervals of difference between group means, are summarized in Figure 4 for mass doubling times and in Figure 5 for cell number doubling times. Tukey’s HSD test confirmed that increasing light intensity from 200 μmol photons·m^−2^⋅s^−1^–400 μmol photons·m^−2^⋅s^−1^ had no significant effect on growth rate (mass doubling time) in *D. quadricauda* and *C. reinhardtii* (Figure 4A). D_2_O concentration significantly affected mass doubling time in all combinations tested, although the difference between 0% D_2_O and 70% D_2_O was the least significant in *D. quadricauda* (Figure 4B). In terms of cell number doubling time, only the light intensity of 100 μmol photons·m^−2^⋅s^−1^ in *C. reinhardtii* showed a significant difference compared with the other tested light intensities (Figure 5A). Similarly, the only concentration of D_2_O that showed a significant difference compared to the other tested variants was 90% D_2_O (Figure 5B). The highest D_2_O concentration proved to be significantly different for both *C. reinhardtii* and *D. quadricauda*.

**TABLE 1:** Mass and cell doubling times (DT) of *C. reinhardtii* and *D. quadricauda* in different combinations of D_2_O and light intensities. Values are given as mean ± standard deviation (*n* = 3).

D_2_O conc. (%)	Light intensity (μmol·m^−2^·s^−1^)	*C. reinhardtii*		*D. quadricauda*	
		Mass DT (h)	Cell number DT (h)	Mass DT (h)	Cell number DT (h)
0	100	20.23 ± 1.37	13.60 ± 0.92	10.27 ± 0.06	10.60 ± 0.43
	200	10.83 ± 0.14	13.56 ± 0.43	7.90 ± 0.01	7.58 ± 0.31
	400	7.77 ± 0.01	8.23 ± 0.20	7.43 ± 0.01	6.98 ± 0.27
70	100	54.77 ± 1.91	55.94 ± 8.50	15.27 ± 0.85	14.77 ± 0.53
	200	32.16 ± 3.62	25.27 ± 2.18	14.75 ± 0.03	14.79 ± 0.48
	400	25.15 ± 0.18	21.59 ± 1.25	13.35 ± 0.41	13.59 ± 0.77
90	100	94.44 ± 2.87	365.61 ± 148.63	61.95 ± 1.39	72.57 ± 22.76
	200	68.87 ± 1.25	121.51 ± 2.30	42.65 ± 2.06	44.17 ± 7.96
	400	72.84 ± 7.91	62.12 ± 11.65	47.45 ± 9.99	62.81 ± 16.52

### Morphology of the cells

**FIGURE 6:**
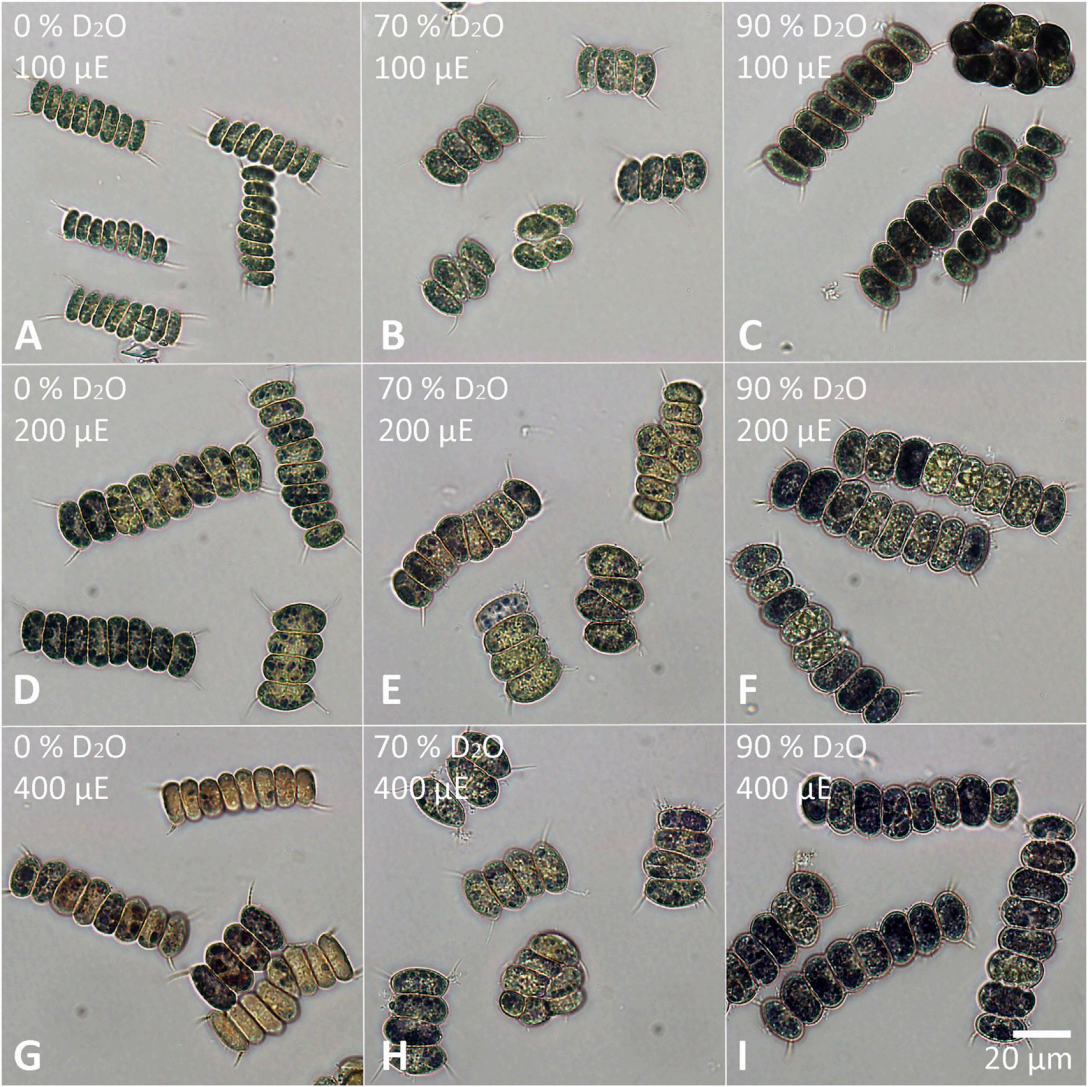
Staining of *D. quadricauda* with Lugol’s solution. Cells grown in 0 **(A,D,G)**, 70 **(B,E,H)** or 90% **(C,F,I)** D2O (from left to right) at an incident light intensity of 100 **(A,B,C)**, 200 **(D,E,F)** or 400 **(G,H,I)** μmol photons⋅m^−2^⋅s^−1^ (denoted as μE in the figure; from top to bottom) were stained after 48 h of cultivation. The starch granules appear dark purple to black.

**FIGURE 7:**
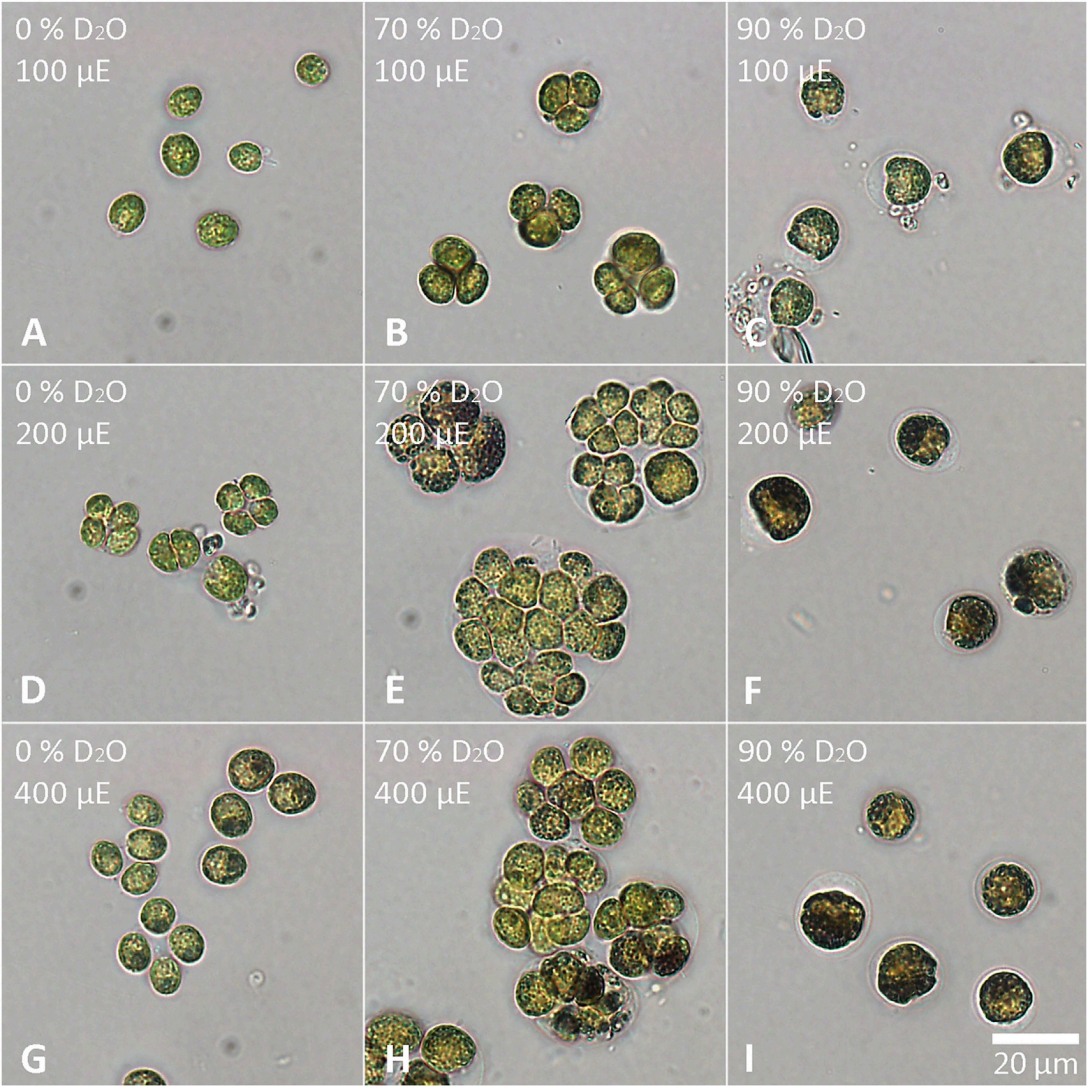
Staining of *C. reinhardtii* with Lugol’s solution. Cells grown in 0 **(A,D,G)**, 70 **(B,E,H)** or 90% **(C,F,I)** D2O (from left to right) at an incident light intensity of 100 **(A,B,C)**, 200 **(D,E,F)** or 400 **(G,H,I)** μmol photons⋅m^−2^⋅s^−1^ (denoted as μE in the figure; from top to bottom) were stained after 48 h of cultivation. The starch granules appear dark purple to black.

**FIGURE 8:**
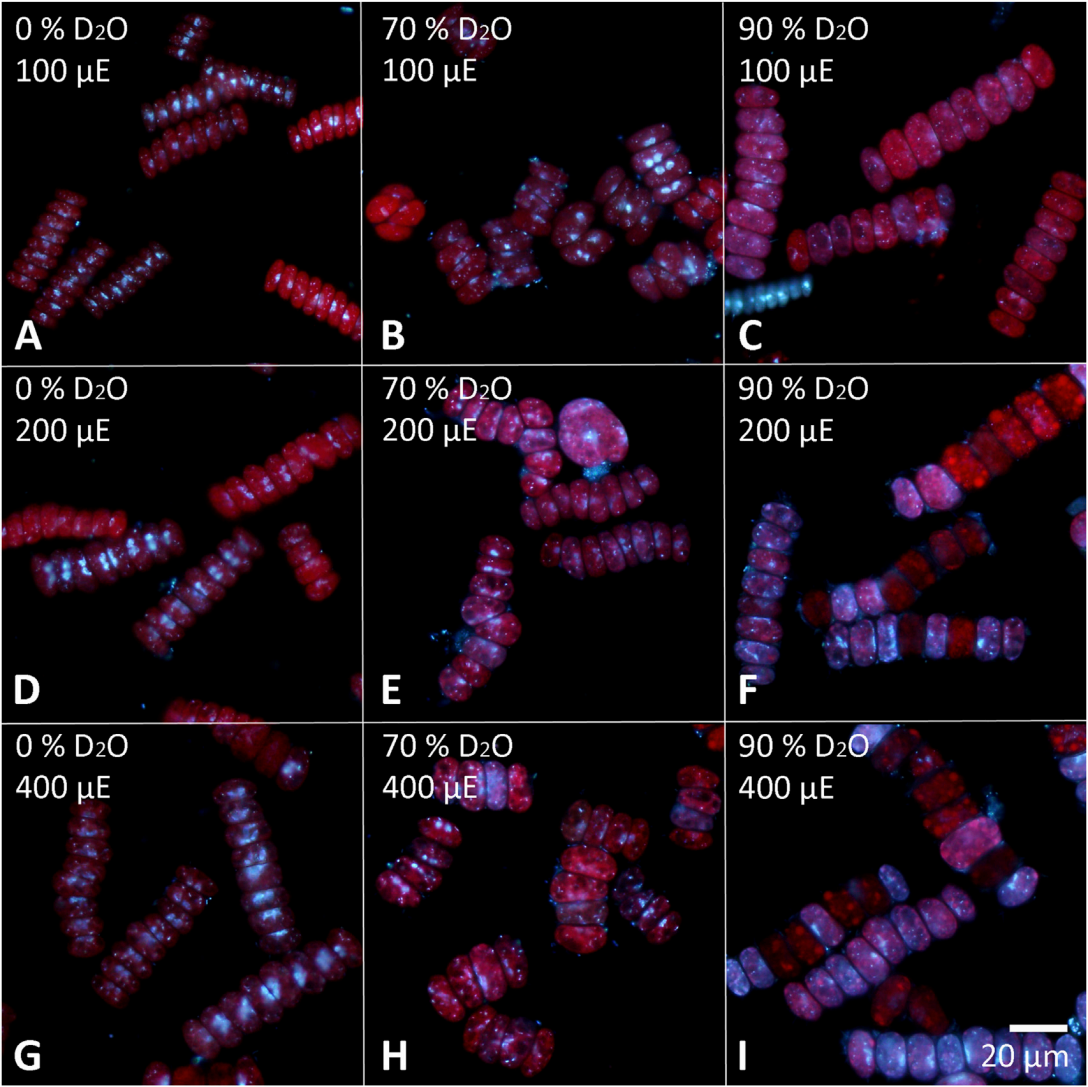
Cells of *D. quadricauda* stained with DAPI to visualize the nuclei. Cells cultured in 0 **(A,D,G)**, 70 **(B,E,H)** or 90% **(C,F,I)** D2O (from left to right) at an incident light intensity of 100 **(A,B,C)**, 200 **(D,E,F)** or 400 **(G,H,I)** μmol photonsm^−2^s^−1^ (denoted as μE in the figure; from top to bottom) were stained after 48 h of cultivation.

**FIGURE 9:**
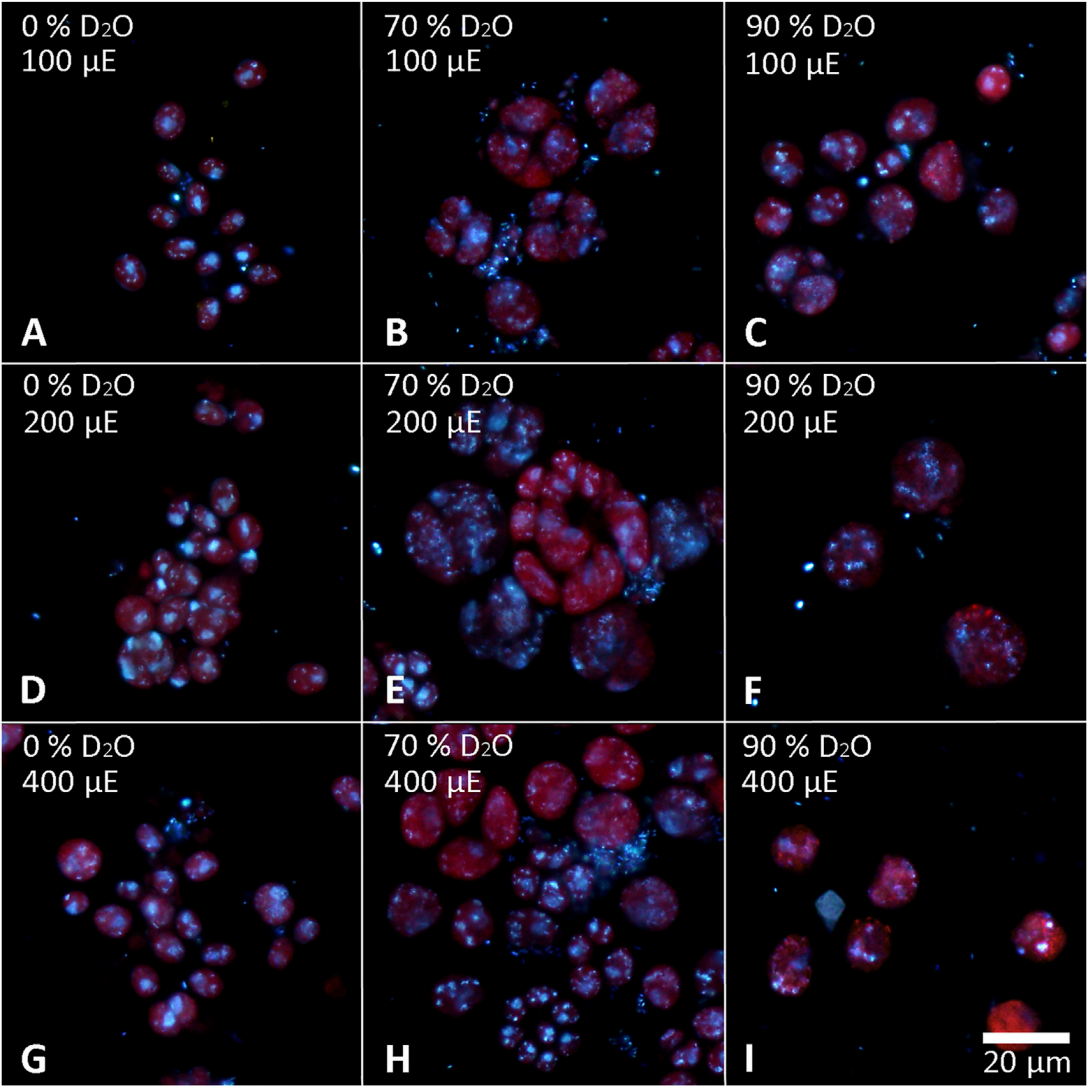
Cells of *C. reinhardtii* stained with DAPI to visualize the nuclei. Cells cultured in 0 **(A,D,G)**, 70 **(B,E,H)** or 90% **(C,F,I)** D2O (from left to right) at an incident light intensity of 100 **(A,B,C)**, 200 **(D,E,F)** or 400 **(G,H,I)** μmol photons⋅m^−2^⋅s^−1^ (denoted as μE in the figure; from top to bottom) were stained after 48 h of cultivation.

The cells of each experimental culture were sampled after 48 h and examined under a light microscope to assess their morphology as well as the presence of starch (Figures 6, 7). Furthermore, the cells were stained with DAPI to analyze the morphology and number of their nuclei (Figures 8, 9). The Lugol´s staining showed presence of starch in the cells of all experimental cultures, with cultures in 90% D_2_O tending to accumulate more visible starch granules (Figures 6C, F, I and 7C, F, I). The morphology of the cells reflected their cell cycle progression. At the time the photographs were taken, all control cultures of *D. quadricauda* had divided at least once, forming mostly eight-celled coenobia (Figures 6A, D, G) with two nuclei visible in most cells, although four nuclei were occasionally present at the highest light intensity (Figures 8A, D, G). In 70% D_2_O, *D. quadricauda* cells also divided at least once at all incident light intensities, but they formed a mixture of eight and four-celled coenobia at 200 and 400 μmol photons·m^−2^⋅s^−1^ and exclusively four-celled coenobia at 100 μmol photons·m^−2^⋅s^−1^ (Figures 6B, E, H). The quality of the nuclear staining appeared to be slightly lower, but at the lowest light intensity, most cells clearly had two nuclei as in the control cultures (Figure 8B). At 200 μmol photons·m^−2^⋅s^−1^ and 400 μmol photons·m^−2^⋅s^−1^, DAPI stained mostly one or two nuclei within the cells and many nucleoids within the chloroplast of the cells’ (Figures 8E, H). The cells of *D. quadricauda* in 90% D_2_O apparently did not divide during the first 48 h of cultivation, regardless of light intensity. Consequently, they showed increased starch accumulation (Figures 6C, F, I). Their nuclei were only weakly stained with DAPI. We observed dying cells with fragmented chloroplasts that showed little to no auto-fluorescence of chlorophyll (Figures 8C, F, I). *C. reinhardtii* cells from the control culture showed normal morphology with intermittent starch content at all light intensities. At the time of observation (48 h), they all underwent at least 1 cell division, mainly giving rise to four daughter cells (Figures 7A,D,G) with a single nucleus and multiple nucleoids (Figures 9A,D,G). *C. reinhardtii* cells grown in 70% D_2_O also completed at least 1 cell division in 48 h and produced four daughter cells. The cells cultured at 200 and 400 μmol photons·m^−2^⋅s.^−1^ formed multicellular structures resulting from successive rounds of cell division without hatching daughter cells (Figures 7B, E, H, 9E). The cells were generally enlarged, with the signal of a single nucleus being somewhat diffuse, especially in larger cells (Figures 9B, E, H). All *C. reinhardtii* cultures in 70% D_2_O contained starch, as shown by staining (Figures 7B, E, H). *C. reinhardtii* cells in 90% D_2_O were enlarged but showed no signs of cell division at the time of observation (48 h). Cells typically contained a single nucleus, which emitted a slightly diffuse signal, and multiple nucleoids (Figures 9C, F, I). The intensity of staining with Lugol’s solution indicated a high starch content (Figures 7C, F, I).

**FIGURE 10:**
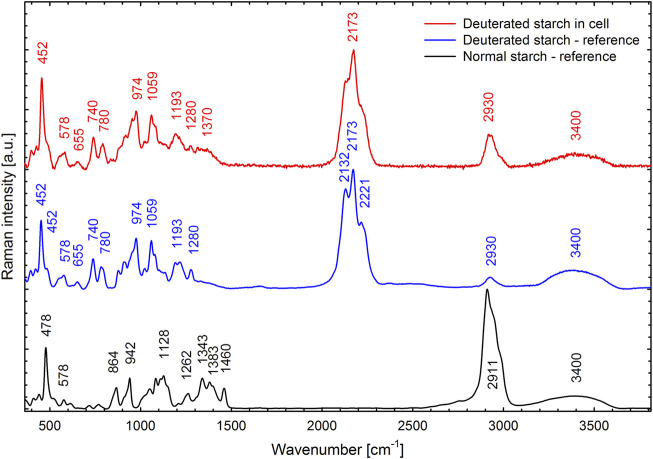
Raman spectrum of deuterated starch found in *C. reinhardtii* cells cultivated in the 90% D_2_O medium for 4 days (red line) compared with a reference spectrum of highly deuterated starch extracted from the *C. reinhardtii* culture cultivated in 99% D_2_O (blue line) and with a normal starch (black line). For all three specimens, the cells or starch granules were dispersed in H_2_O. Spectral contribution of H_2_O was subtracted and the background was corrected.

**FIGURE 11:**
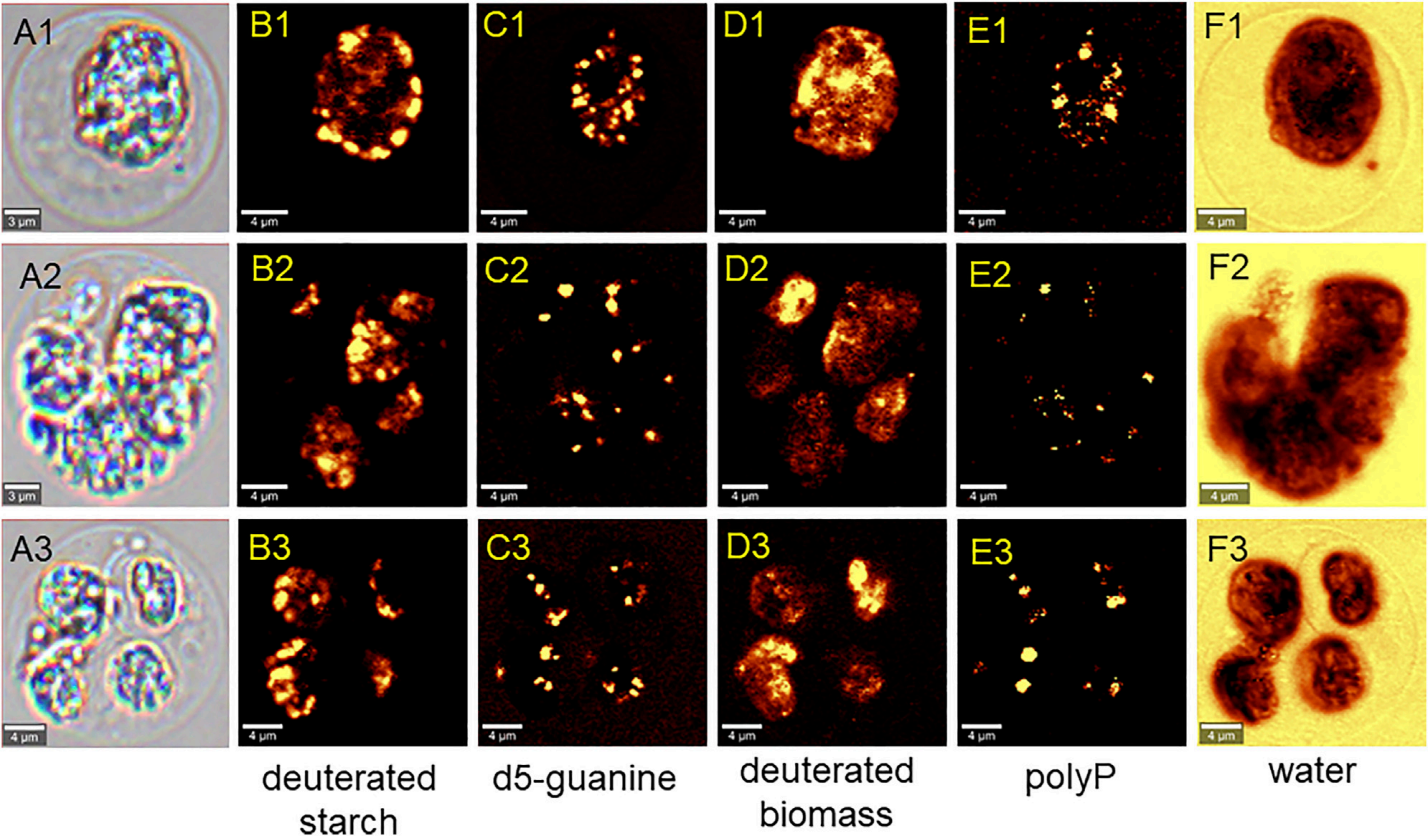
Raman chemical maps of three representative *C. reinhardtii* cells cultivated in the 90% D_2_O for 4 days. In the cells, spatial distribution of deuterated starch (B1–B3), crystalline deuterated d5-guanine (C1–C3), deuterated general biomass (D1–D3), polyphosphates (E1–E3) and water (F1–F3) is shown.

The cells that grew in 70% D_2_O increased their cell number by 2-fold to more than 200-fold during the experiment (Figure 3B) and therefore clearly had to contain deuterated biomolecules. For the cultures that grew in 90% D_2_O and did not divide, the situation was not clear (Figure 3C). Therefore, the presence of deuterated compounds in the cells was checked under the confocal Raman microscope. Raman microscopy was able to distinguish not only the individual biomolecules but also their deuteration. The high similarity of the Raman spectrum of starch in the cells transferred to 90% D_2_O medium with the reference spectrum of highly deuterated starch indicated a high degree of deuteration (Figure 10). The most important Raman marker of starch deuteration is the intensity ratio of the broad bands of C-D and C-H stretching vibrations centered at 2173 and 2911 cm^−1^, respectively. According to this ratio, a large fraction of hydrogens covalently bonded to carbon is replaced by deuterium. A small band at 2930 cm^−1^ indicated a small contribution from C-H groups, but the reference spectrum of highly deuterated starch also shows a small contribution from hydrogen bonds. The broad bands centered at 3400 cm^−1^ belong to O-H stretching vibrations of hydroxyl groups of starch, which are accessible to the solvent and can be rapidly exchanged by protium/deuterium depending on the nature of the solvent. Close examination of the individual cells showed that the entire cell biomass was deuterated (Figures 11D1–3) with clearly deuterated starch (Figures 11B1–3) and guanine crystals (Figures 11C1–3). The cells also contained polyphosphates as another form of energy reserve (Figures 11E1–3). The Raman spectra of each component can be found in Supplementary Figure S1.

### Cellular stress

**FIGURE 12:**
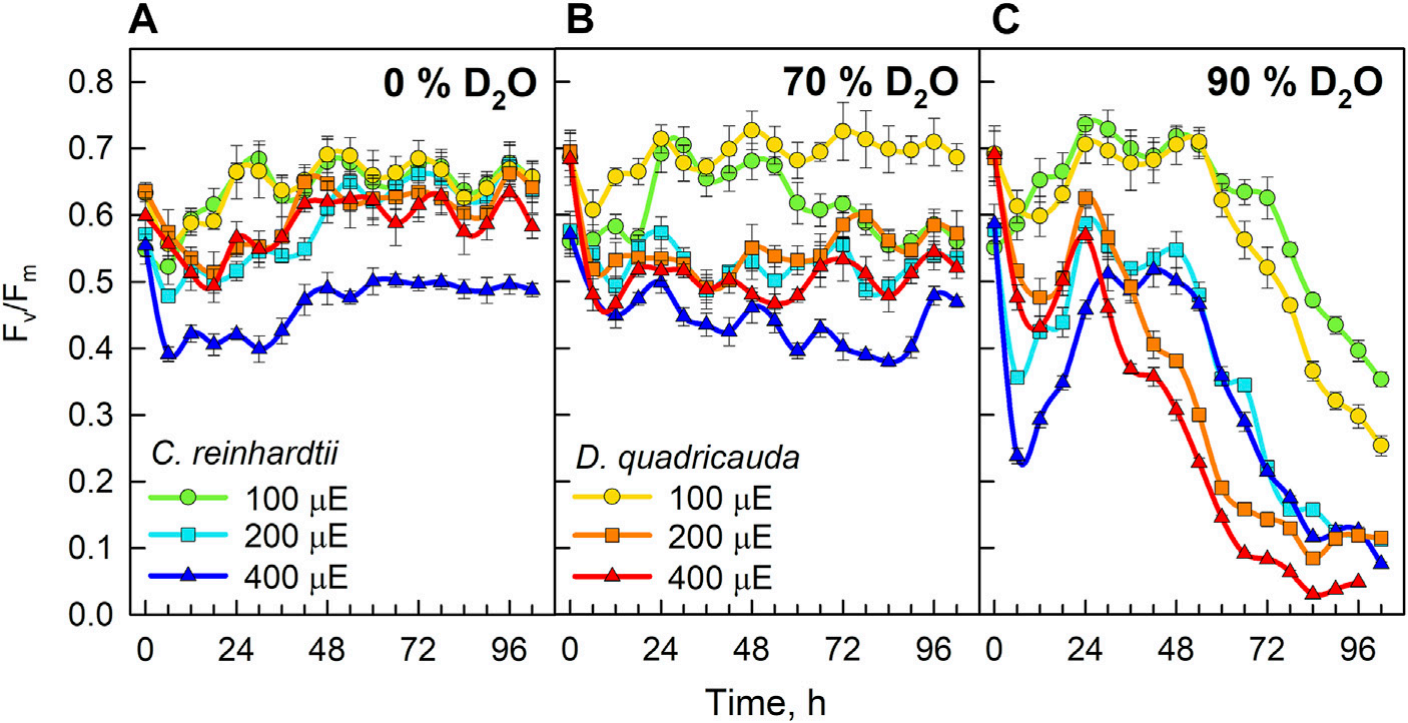
F_v_/F_m_ ratio of cultures grown in 0% D_2_O **(A)**, 70% D_2_O **(B)** or 90% D_2_O **(C)** at three light intensities 100, 200, and 400 μmol photons·m^−2^⋅s^−1^. Light intensities are given in μE (instead of μmol photons·m^−2^⋅s^−1^), in order to fit the legends.

To measure the cellular stress caused by the experimental treatment, measurements of chlorophyll fluorescence were used to estimate the stress acting on the photosynthetic machinery (Figure 12). All control cultures, except *C. reinhardtii* at 400 μmol photons·m^−2^⋅s^−1^, showed a decrease in F_v_/F_m_ during the first 12 hours. Thereafter, F_v_/F_m_ recovered and fluctuated between 0.58 and 0.7 throughout the experiment. In *C. reinhardtii* at 400 μmol m^−2^s^−1^, F_v_/F_m_ dropped to 0.4 during the first 24 h and then recovered to approximately 0.5 until the end of the experiment (Figure 12A). Cultures grown in 70% D_2_O showed a drop in F_v_/F_m_ similar to control cultures at the beginning of the experiment. However, complete recovery of F_v_/F_m_ was observed in both *C. reinhardtii* and *D. quadricauda* only in cultures grown at the lowest light intensity. Cultures grown in 70% D_2_O at light intensities of 200 and 400 μmol photons·m^−2^⋅s^−1^ recovered only partially, with the highest F_v_/F_m_ observed in *D. quadricauda* at 200 μmol photons·m^−2^⋅s^−1^. Moreover, it was evident that with increasing light intensity, the observed F_v_/F_m_ values decreased in both *D. quadricauda* and *C. reinhardtii* (Figure 12B). This trend was even more pronounced in 90% D_2_O, where light intensities of 400 and 200 μmol photons·m^−2^⋅s^−1^ caused a dramatic decrease in F_v_/F_m_, indicating severe stress in both *D. quadricauda* and *C. reinhardtii*. At a light intensity of 100 μmol photons·m^−2 ^⋅s^−1^, both organisms maintained F_v_/F_m_ at approximately 0.7 for the first 48 h, but then F_v_/F_m_ decreased to 0.25 and 0.35 for *D. quadricauda* and *C. reinhardtii*, respectively (Figure 12C).

**FIGURE 13:**
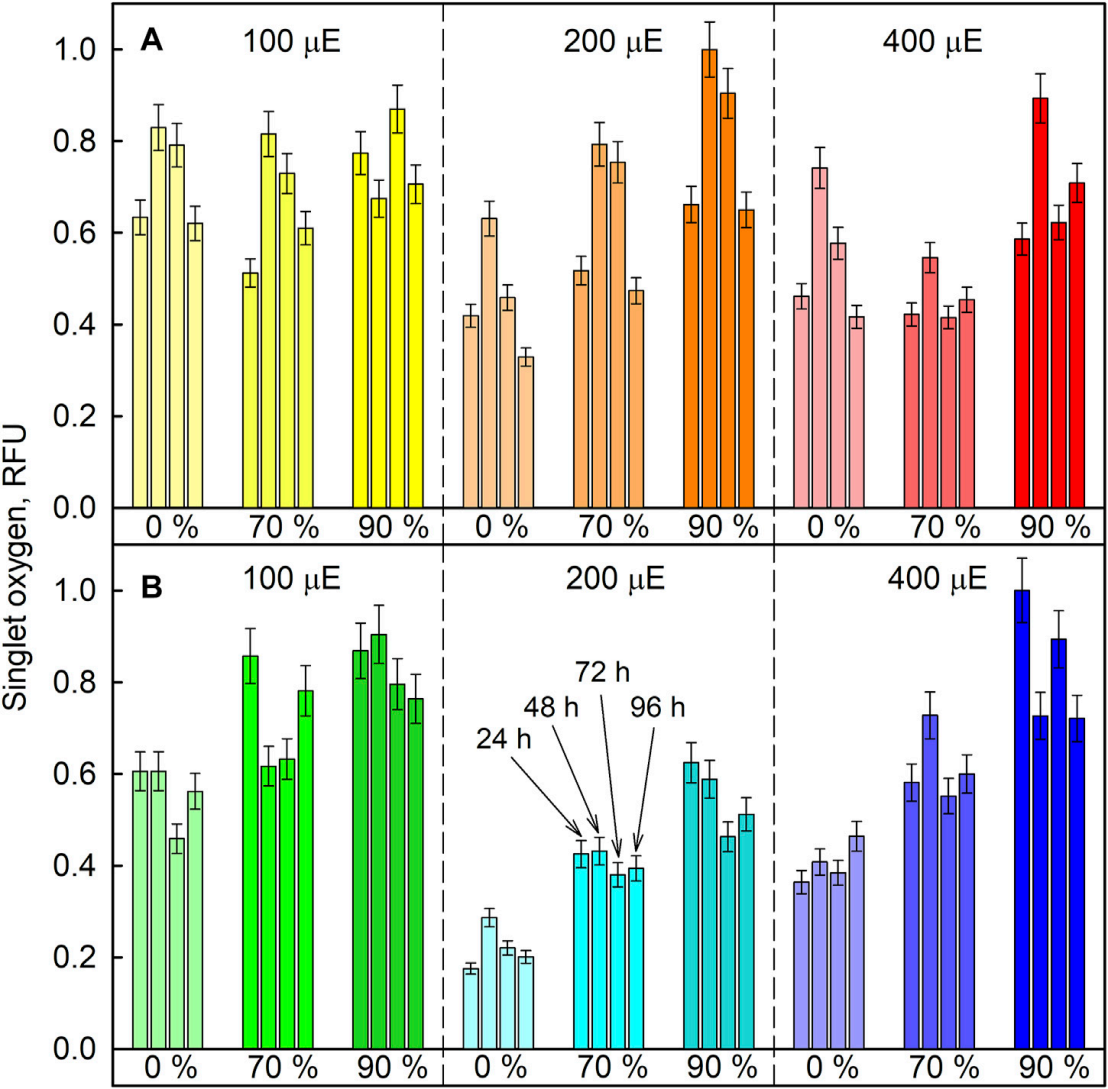
Relative amount of singlet oxygen in cultures grown at different incident light intensities (100, 200, and 400 µmol photons·m^−2^⋅s^−1^) and at different D_2_O concentrations (0%, 70% and 90%, indicated as percentages under the bars) in *D. quadricauda* **(A)** and *C. reinhardtii* **(B)**. Singlet oxygen levels were determined spectrophotometrically and fluorescence was normalized to the maximum value. Individual bars within one group (same color) represent individual measurements after 24, 48, 72, and 96 h of cultivation (as indicated in panel B). Light intensities are given in μE (instead of µmol photons·m^−2^⋅s^−1^), in order to fit the legends.

**FIGURE 14:**
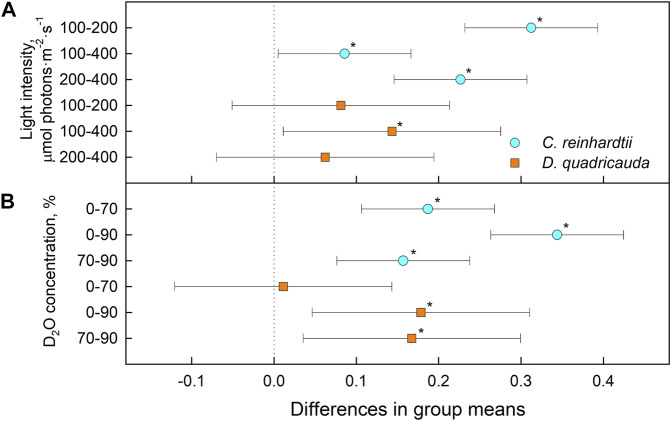
Tukey’s HSD test of singlet oxygen levels plotted as 95% confidence intervals of mean differences between groups indicated on the left. The effect of light intensity **(A)** and the effect of D_2_O concentration **(B)** on singlet oxygen levels in *C. reinhardtii* (cyan circles) and *D. quadricauda* (orange squares) are shown separately. A difference in group means (X axis) equal to zero indicates that the group means are equal, so only a confidence interval that does not contain zero is statistically significant (*p* = 0.05, indicated by an asterisk).

The relative levels of singlet oxygen were used to estimate oxidative stress caused by the experimental treatment. All cultures were sampled after 24, 48, 72, and 96 h of cultivation, and singlet oxygen levels were determined and compared between experimental treatments in *D. quadricauda* (Figure 13A) and *C. reinhardtii* (Figure 13B). While it is clear that singlet oxygen levels in a culture varied over the course of the experiment, there was also a trend toward higher singlet oxygen levels at higher D_2_O concentrations, particularly in *C. reinhardtii* (Figure 13B). A two-way ANOVA revealed that there was no statistically significant interaction between the effects of light intensity and D_2_O concentration on singlet oxygen levels in either *C. reinhardtii* [F (4,27) = 1.053, *p* = 0.399) or *D. quadricauda* (F (4,27) = 1.978, *p* = 0.126]. However, a simple main effects analysis showed that both light intensity and D_2_O concentration individually had a statistically significant effect on singlet oxygen levels in *C. reinhardtii* (both with *p* < 0.001) as well as in *D. quadricauda* (*p* = 0.040 and *p* = 0.003, respectively). The differences between individual light intensities and D_2_O concentrations were evaluated using Tukey’s HSD test, and the 95% confidence intervals of the mean differences between groups are shown in Figure 14. The differences in singlet oxygen levels between all tested light intensities in *C. reinhardtii* were statistically significant, but only the difference between the lowest and highest light intensity in *D. quadricauda* was significant (Figure14A). The differences in singlet oxygen levels between all tested D_2_O concentrations were statistically significant in both *C. reinhardtii* and *D. quadricauda*, except for the comparison of 0% D_2_O and 70% D_2_O, which showed no statistically significant difference in singlet oxygen levels in *D. quadricauda* (Figure 14B).

## Discussion

### Growth and division

Light intensity is a crucial factor affecting biomass yield in microalgal cultivation. However, biomass yield can also be affected by other factors, such as nutrient availability, temperature or CO_2_ levels (Lee et al., 2015). Therefore, it is important to identify the saturating light intensity for each organism in a given cultivation setup. Monitoring the optical density of cultures (OD_750_) in different combinations of deuterated media and incident light intensities led to the estimation of the saturating light intensity in such cultures. Comparing the performance of the control cultures of *D. quadricauda* and *C. reinhardtii* at different light intensities (Figure 2A; Table 1), it was clear that the *D. quadricauda* cultures in this cultivation setup were saturated at 200 μmol photons·m^−2^⋅s^−1^, as a further increase in light intensity did not significantly improve culture growth. *C. reinhardtii*, on the other hand, was saturated at incident light intensities of 400 μmol photons·m^−2^⋅s^−1^ or higher (Figure 2A; Table 1). Data from higher light intensities are required to confirm that 400 μmol photons·m^−2^⋅s^−1^ is sufficient to fully saturate such a culture. These results are consistent with the published results of Bialevich et al. (2022), who showed a saturation light intensity of 500 μmol photons·m^−2^⋅s^−1^ or more for *C. reinhardtii* and 200 μmol photons·m^−2^⋅s^−1^ for *D. quadricauda* in the same cultivation setup. Cultures of *D. quadricauda* in 70% D_2_O were saturated even at the lowest incident light intensity tested (Figure 2B; Table 1), indicating that D_2_O is the main stressor affecting cell physiology regardless of light intensity. *C. reinhardtii* in 70% D_2_O showed increasing performance with increasing light intensity, confirming that saturating light intensity was generally higher than in *D. quadricauda*, even in deuterated medium (Figure 2B; Table 1). Growth of cultures in 90% D_2_O was affected primarily by the presence of deuterium, which greatly retarded growth regardless of incident light intensity (Figures 2C, 4, 5; Table 1). A reduction in saturating light intensity or higher sensitivity to light under suboptimal stress conditions has been previously described for salinity or high temperature stress (León and Galván, 1999; Possmayer et al., 2011; Annan, 2014). However, the apparent growth of the culture could be caused (at least in the short term) by two interrelated cellular processes: growth in size and cell division. While the increase in optical density could be caused by either of these factors, the increase in cell number reflects only the ability of cells to divide. The increase in cell number of cells grown in 0% D_2_O largely reflected their increase in optical density, indicating normal progression of the cell cycle in which the increase in cell volume is followed by cell division (Figure 3A; Table 1). Cells in 70% D_2_O were generally capable of cell division, as evidenced by the increase in cell number (Figure 3B), although the time required for cell division was longer compared with the control culture at the same light intensity (Figure 3B; Table 1). In addition, the number of daughter cells formed within 1 cell cycle tended to be lower in 70% D_2_O for both *D. quadricauda* and *C. reinhardtii* (compare 0% D_2_O and 70% D_2_O in Figures 6, 7). A reduced number of daughter cells is a typical sign of suboptimal growth conditions or stress in algae dividing by multiple fission and has already been described for *C. reinhardtii* and *Parachlorella kessleri* grown in medium containing D_2_O (Zachleder et al., 2021a; Kselíková et al., 2021). Cultures grown in 90% D_2_O showed barely any increase in cell number regardless of light intensity (Figures 3C, 5; Table 1). Therefore, cell division was severely delayed in both *D. quadricauda* and *C. reinhardtii* in 90% D_2_O. These results are consistent with previously published results in *C. reinhardtii* (Kselíková et al., 2021), *Parachlorella kessleri* (Zachleder et al., 2021a; Zachleder et al., 2021b) or *Chlorella ellipsoidea* (Unno et al., 1992). Interestingly, Tukey’s HSD test revealed that while both mass and cell number doubling times were affected by deuterium treatment (Figures 4, 5), the difference between 0% D_2_O and 70% D_2_O was statistically significant only with respect to cell number doubling time. This clearly indicates differing sensitivities of cell growth (reflected in mass doubling time) and cell division (reflected in cell number doubling time) to deuterium treatment, especially at intermediate D_2_O concentrations. At the same time, a statistically significant difference in mass and cell number doubling time between 70 % and 90% D_2_O proves a concentration-dependent delay in growth (and division) in both *C. reinhardtii* and *D. quadricauda*. Therefore, careful selection of the appropriate D_2_O concentration is of utmost importance if algal cultures are to be used for the production of deuterated compounds, especially with respect to production goals. No less attention should be paid to the choice of organism, as our data show that *C. reinhardtii* is more sensitive to deuterium for all monitored parameters (Table 1; Figures 4, 5, 12).

### Cell morphology

Cell growth in microalgae is associated with the accumulation of energy-storing molecules that are catabolized later in the cell cycle to support cell division processes (Zachleder et al., 2016; Torres-Romero et al., 2019; Ivanov et al., 2021). Both microalgae tested store energy for cell division mainly in the form of starch (Figures 6, 7, 11), no lipids were detected (data not shown). Since cell division is impaired in deuterated medium, both *D. quadricauda* (Figure 6) and *C. reinhardtii* (Figure 7) cells were noticeably enlarged. In addition, cells in deuterated medium, especially in 90% D_2_O, contained significant amount of starch granules, as shown by Lugol’s staining (Figures 6C, F, I, 7C, F, I) as well as by confocal Raman microscopy (Figures 11B1–3). The same trend was observed in other microalgae after treatment with deuterium (Zachleder et al., 2021a; Kselíková et al., 2021), supraoptimal temperature (Zachleder et al., 2019; Zachleder et al., 2021b) or nutrient starvation (Jerez et al., 2015). All of the above treatments have one thing in common—they impair/delay cell division, so starch accumulation is in fact a consequence of cell cycle arrest/slowdown. To further investigate cell cycle impairment in deuterated cultures, we performed DAPI staining to assess the ability of cells to divide their nuclei, as DNA replication and nuclear division are among the most sensitive processes of cell cycle. Both *D. quadricauda* and *C. reinhardtii* grown in 70% D_2_O divided their nuclei (Figures 8B, E, H, 9B, E, H), whereas in 90% D_2_O nuclei division was less common (Figures 8C, F, I, 9C, F, I). However, nuclear division, although negatively affected, is not the only obstacle to complete the cell cycle in highly deuterated cultures. The presence of large binuclear cells without any sign of protoplast fission (e.g., Figures 9C,F,I) suggests that other division-related processes may be the bottleneck for complete cell division in highly deuterated cultures. In addition, cells in deuterated cultures of *C. reinhardtii* showed evidence of defective cell hatching after division, as documented by the appearance of multicellular division clusters or palmelloids that remained intact for extended periods of time (Figures 7, 9). The formation of palmelloids is a mechanism for coping with stress in *C. reinhardtii* (Khona et al., 2016; de Carpentier et al., 2019), further highlighting the effect of deuterium as a stress-inducing agent. Also noteworthy is the morphology of chloroplasts in 90% D_2_O, as seen under fluorescence microscopy (Figures 8C, F, I, 9C, F, I). Compared with control cells or even cells in 70% D_2_O, cells in 90% D_2_O showed uneven chlorophyll auto-fluorescence, as if their chloroplasts were fragmented. Structural changes in chloroplasts in response to various environmental stresses have been described previously in algae (Poppe et al., 2002; Zuppini et al., 2007). They are also directly related to oxidative stress in chloroplasts (Woodson, 2022), as has also been observed in deuterated cultures (see below).

### Cellular stress

The different physicochemical properties of deuterium oxide compared to normal water include not only differences in boiling and freezing points, but also in density and viscosity (Yang, 2016), and affect the function of deuterium oxide as a solvent in biological systems. The larger mass of deuterium leads to the formation of stronger bonds and increases the dissociation and activation energy of bonds. In addition to increasing the energy demand of cells, deuterium also decreases the energy pool (Vasilescu and Katona, 1986) by affecting ATP synthase (Kotyk et al., 1990; Olgun, 2007), the mitochondrial respiratory chain (Salomonsson et al., 2008), and photosynthesis (de Kouchkovsky et al., 1982). Consequently, the presence of deuterium affects overall metabolism at multiple levels. The algal cultures coped well with 70% D_2_O, which allowed division into 2 to more than 200 daughter cells over 96 h, depending on light intensity (Figure 3B). The daughter cells formed contained biomass deuterated up to 70% confirming the resistance of algal cells to lower deuterium concentrations (Bhosale et al., 2006; Garg et al., 2017). In 90% D_2_O, both biomass and energy compounds were deuterated (Figures 11B1–D3), although (almost) no cell division occurred (Figure 3C). Given the widespread effect of deuterium on general metabolism, there is likely not a single causative process behind the observed deuterium effect. Nevertheless, it may be interesting to narrow down the differences in response to 70% and 90% D_2_O by combining transcriptomics gene expression analyses guiding metabolomics. Irrespective of the mechanism, deuteration induced a complex stress response in the microalgae tested. Therefore, we chose to measure the F_v_/F_m_ ratio, which represents the maximum potential quantum efficiency of photosystem II, when all reaction centers are open (Figure 12). F_v_/F_m_ is a widely used indicator of stress affecting photosystem II. Under stress conditions, photoprotective mechanisms are active, thus effectively reducing the maximum efficiency of photosystem II. The majority of the cultures, including the control cultures, showed a light-dependent decrease in the Fv/Fm ratio at the beginning of cultivation. This could be due to the initial adaptation of the diluted synchronous culture to the sudden incidence of light immediately after dark treatment. Such a drop is not always the rule for synchronous cultures under similar culture conditions. The control cells eventually adapted to the new conditions and recovered from the drop. Deuterium treatment decreased F_v_/F_m_ values as expected (Figure 12). In addition, the extent of the F_v_/F_m_ decrease was largely influenced by light intensity, with higher light intensity generally further decreasing F_v_/F_m_ values (Figures 12B,C). Moreover, F_v_/F_m_ values appeared to be stabilized in 90% D_2_O for about 48 h (24 h at the highest light intensity), and then dropped rapidly, reflecting the overall decreasing fitness of cells under harsh stress conditions (Figure 12C). However, higher stress levels, determined by F_v_/F_m_ measurements in deuterated cultures at higher light intensities, did not result in poorer culture performance, as higher light intensities resulted in better culture growth, both in terms of cell mass and cell numbers, for both organisms tested under control conditions and for *C. reinhardtii* even in 70% D_2_O (Figures 2, 3). Because the maximum potential quantum efficiency of photosystem II was reduced in deuterated cultures, we wanted to determine whether excess light could cause oxidative stress in cells. In situations where the absorbed light exceeds the capacity of photosynthesis, singlet oxygen could be generated via the formation of triplet chlorophyll (Figure 13). Singlet oxygen is normally detoxified by the antioxidant machinery of cells, otherwise it triggers the upregulation of genes involved in the response to photo-oxidative stress (Krieger-Liszkay, 2005). In addition, deuterium has been found to increase the lifetime of singlet oxygen due to solvent isotope effects (Kajiwara and Kearns, 1973). In our data set, singlet oxygen generally increased with increasing D_2_O concentration, except for *D. quadricauda* grown in 0% D_2_O and 70% D_2_O, where no statistically significant differences in singlet oxygen levels were observed (Figures 13, 14B). The effect of light intensity on singlet oxygen level was significantly different in *C. reinhardtii* at all intensities tested (Figure 14A), but only the lowest and highest light intensities resulted in a statistically significant difference in *D. quadricauda* (Figure 14A). Higher levels of singlet oxygen could contribute to overall lower fitness of cells grown in D_2_O by causing photo-oxidative damage or by playing a role in chloroplast degradation (Fischer et al., 2006; Erickson et al., 2015; Woodson, 2022).

## Conclusion

In the present work, we investigated the effects of light intensity and deuterated water in the culture medium on growth and division of microalgae *Desmodesmus quadricauda* and *Chlamydomonas reinhardtii*. Our results show a significant slowdown in culture growth in deuterated medium, with division processes being more affected than cell growth. Impaired cell division processes in highly deuterated medium lead to enlargement of cells and the accumulation of energy-storing molecules, such as starch, that would normally be consumed to support cell division processes. By combining growth in deuterated medium with the use of different light intensities for cultivation, we were able to determine the saturating light intensity for both organisms under all conditions tested. The saturating light intensity of the deuterated cultures was significantly lower because the stress caused by deuteration is enhanced by strong light, as evidenced by an increase in singlet oxygen, which can contribute to photooxidative damage, and an upregulation of photoprotection mechanisms and subsequent decrease in the F_v_/F_m_ ratio. Increased photosensitivity associated with deterioration of photosynthetic performance at high deuterium concentrations is apparently part of the general stress response induced by deuterium and its strong KIE. Although such findings may discourage further attempts to culture microalgae in deuterated medium, it should be noted that the D_2_O concentrations used in this study were very high to elucidate a response of model organisms to extreme environmental conditions and to set the limits of possible use of deuterium in microalgal biotechnology. A higher concentration of deuterium in the growth medium led to a rapid and high degree of labelling, but also negatively affected the growth of the culture. On the other hand, a lower deuterium concentration, which had less impact on cell cycle progression, may be more than sufficient to produce deuterated compounds with a satisfactory degree of labelling. Moreover, the type of deuterated compound required plays a crucial role in the design of experiments, as the production of some compounds is not linked to the ability of cells to complete their cell cycle or may even be enhanced when cell cycle progression is blocked, particularly energy-storing molecules such as starch, lipids, or polyphosphates (Yao et al., 2012; Zachleder and Brányiková, 2014; Vitova et al., 2015; Moudříková et al., 2016). Moreover, different organisms show differing sensitivities to deuterium, as shown by our data, where *C. reinhardtii* was more sensitive to deuterium for all monitored parameters. Therefore, we believe that proper culture management can exploit the potential of microalgae in the production of deuterated biomolecules. Although the D_2_O used to produce the growth media is expensive (currently over 1000 EUR/litre), deuterated molecules are of even higher value and their biological production is a viable process that is cheaper than chemical synthesis (Zachleder et al., 2018). Moreover, D_2_O used in the growth media might be partly re-used in further rounds of cultivation if properly purified. We have shown that cultures grown in 70% D_2_O in the growth medium can maintain adequate growth and cell fitness while producing deuterated biomolecules. If the goal is to obtain biomolecules with a higher degree of deuteration, another option might be to transfer synchronous cultures of microalgae in the early stages of their cell cycle to highly deuterated medium. These cells would grow larger and produce energy-storing molecules. If an appropriate cell density is used as an inoculum, the yield of highly deuterated compounds would overcome both the lower biomass yield and the cost of the two-step cultivation process. However, other factors affecting algal productivity (temperature, medium composition, CO_2_ concentration, etc.) may still prove critical to the successful management of deuterated cultures.

## Data Availability

The original contributions presented in the study are included in the article/Supplementary Material, further inquiries can be directed to the corresponding author.

## Supplementary material

Supplementary Figure S1 Raman spectra of biomolecules identified in *C. reinhardtii* cells cultivated in the 90% D_2_O for 4 days. All the biomolecules containing C-H groups (starch, crystalline guanine, and general biomass covering protein and lipids from plastids) were synthesized *de novo* because they show high degree of deuteration. As anionic polyphosphate does not contain any hydrogen bond, cultivation in D_2_O has no effect on its Raman spectrum. Since pigments were washed out by methanol on the specimen preparation, their Raman spectra were not detected.
