# Supplementary figures and images for: Cultivation of the microalgae Chlamydomonas reinhardtii and Desmodesmus quadricauda in highly deuterated media: Balancing the light intensity
Source: Front Bioeng Biotechnol. 2022 Sep 5;10:960862. doi: 10.3389/fbioe.2022.960862 (PMC9483122; doi:10.3389/fbioe.2022.960862)

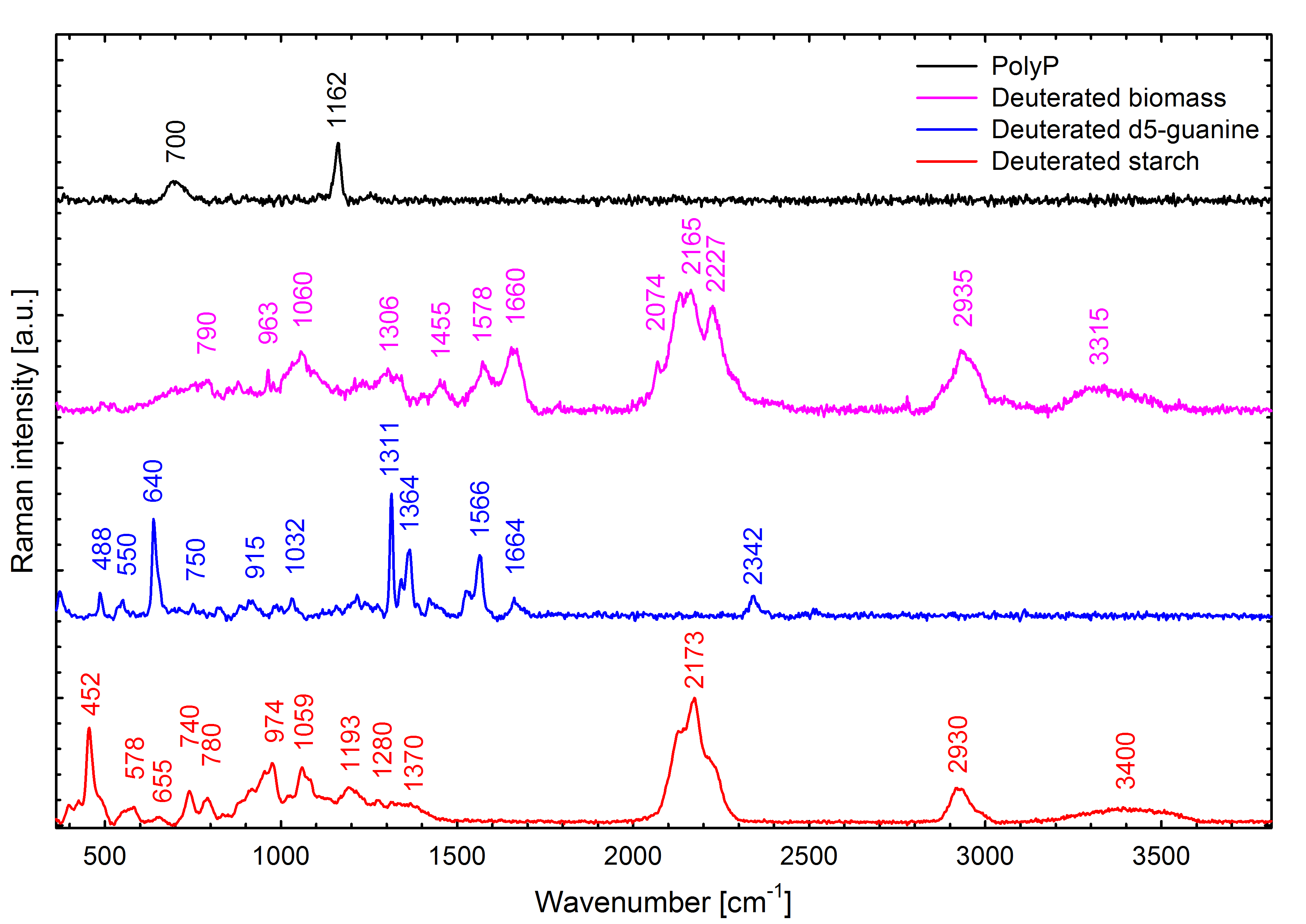

Supplement: Supplementary file 1 [file Image1.TIF]
